# Expression of LIM kinase 1 is associated with reversible G1/S phase arrest, chromosomal instability and prostate cancer

**DOI:** 10.1186/1476-4598-6-40

**Published:** 2007-06-08

**Authors:** Monica Davila, Darshana Jhala, Debashis Ghosh, William E Grizzle, Ratna Chakrabarti

**Affiliations:** 1 Department of Molecular biology and Microbiology, University of Central Florida, Orlando, Florida, USA; 2 Department of Pathology, University of Alabama at Birmingham, Birmingham, Alabama, USA; 3 Department of Biostatistics, University of Michigan, Ann Arbor, Michigan, USA

## Abstract

**Background:**

LIM kinase 1 (LIMK1), a LIM domain containing serine/threonine kinase, modulates actin dynamics through inactivation of the actin depolymerizing protein cofilin. Recent studies have indicated an important role of LIMK1 in growth and invasion of prostate and breast cancer cells; however, the molecular mechanism whereby LIMK1 induces tumor progression is unknown. In this study, we investigated the effects of ectopic expression of LIMK1 on cellular morphology, cell cycle progression and expression profile of LIMK1 in prostate tumors.

**Results:**

Ectopic expression of LIMK1 in benign prostatic hyperplasia cells (BPH), which naturally express low levels of LIMK1, resulted in appearance of abnormal mitotic spindles, multiple centrosomes and smaller chromosomal masses. Furthermore, a transient G1/S phase arrest and delayed G2/M progression was observed in BPH cells expressing LIMK1. When treated with chemotherapeutic agent Taxol, no metaphase arrest was noted in these cells. We have also noted increased nuclear staining of LIMK1 in tumors with higher Gleason Scores and incidence of metastasis.

**Conclusion:**

Our results show that increased expression of LIMK1 results in chromosomal abnormalities, aberrant cell cycle progression and alteration of normal cellular response to microtubule stabilizing agent Taxol; and that LIMK1 expression may be associated with cancerous phenotype of the prostate.

## Background

LIMK1 belongs to a family of unique LIM domain containing serine/threonine kinases (LIMK1 and LIMK2). It modulates actin dynamics by inactivating phosphorylation of cofilin, a member of the ADF (actin depolymerizing factor) family [1,2]. LIMK1 is expressed predominantly in the brain but a modest expression of LIMK1 has been noted in other organs. Recently, a novel role for LIMK1 as an oncogene has been demonstrated in prostate and breast cancer cells [3,4]. LIMK1 is upregulated in malignant prostate tissues compared to histologically normal or benign prostates [3]. Biochemical and in vivo studies have indicated that LIMK1 is involved in promotion and maintenance of the invasive behavior in prostatic and breast cancer cells [3-5] and that when injected in nude mice, breast cancer cells overexpressing LIMK1 are capable of formation of osteolytic lesions in lower extremities and induction of tumor angiogenesis [4,5]. Indirect evidence based on comparative genomic hybridization analysis have indicated a significant correlation between high-stage prostate cancers and chromosomal gains in the short arm of chromosome 7 (7q11.2) [6], which includes the region where LIMK1 is located (7q11.23).

The structural features of LIMK1 include two N-terminal LIM domains in tandem, a single PDZ domain and a C-terminal kinase domain [7,8]. LIMK1 also contains a single nuclear localization signal and a single exit signal [9]. Functional activation of LIMK1 requires phosphorylation at T^508 ^mediated by p21-activated kinase PAK1 and ROCK [10,11]. Activation of LIMK1 through ROCK promotes accumulation of F actin through inactivation of cofilin, a Rho phenotype, which results in formation of stress fibers [11]. Importantly, LIMK1 is also activated by PAK1, which is the effector molecule of the Rac-induced lamellipodia formation or cell spreading [12].

LIMK1 undergoes a distinct pattern of activation during mitosis, becoming hyperphosphorylated and activated at the prometaphase and metaphase and gradually becoming inactivated during telophase and cytokinesis [13,14]. Mitosis-specific activation and phosphorylation of LIMK1 is mediated by Cdk1 [14] but not through active PAK or ROCK [14]. The activation pattern of LIMK1 corresponds to the phosphorylation and dephosphorylation pattern of cofilin during mitosis, which is essential for proper cytokinesis [15]. Thus, maintenance of optimum concentrations and activation of LIMK1 at specific mitotic phases are critical for normal cell cycle progression. Cdk inhibitor p57^Kip ^interacts with LIMK1 and sequesters it to the nucleus [16] as a result of inhibition of Rho signaling by Cip/Kip family members. LIMK1 exhibits contrasting function of activation and inactivation of Cyclin D1 expression depending on the activation of Rac/Cdc42 or Rho-Rho kinase pathways [17]. There also are conflicting reports on expression of Cdk inhibitors (p21^Cip1 ^or p27^Kip1^) following inactivation of Rho-kinase or LIMK1 [18,19]. To date, it is uncertain whether an active LIMK1 is essential for progression of cells through the G1/S phase. The other family member, LIMK2, also is activated through ROCK at T^505 ^[20]; and it is involved in meiosis during maturation of Xenopus oocyte through phosphorylation of cofilin [21]. Activation of ROCK also stimulates cell cycle progression partly through increased cyclin A levels via LIMK2 [22]. Nonetheless, LIMK1 and LIMK2 exhibit different cellular functions and subcellular localizations [23,24].

We have noted that LIMK1 but not LIMK2 is overexpressed in highly aggressive and metastatic PC3 prostate cancer cells and in prostate tumor tissues compared to benign prostatic hyperplasia (BPH-1) cells and normal prostatic epithelium. However, the precise role of LIMK1 in development of abnormal cellular processes, which might facilitate prostate tumor growth and behavior, is unclear. In this study, we demonstrate that increased expression of LIMK1 is associated with accumulation of chromosomal abnormalities, and development of cell cycle defects in cells that naturally express lower concentrations of LIMK1. We also show that expression of LIMK1 is higher in prostate tumors with higher Gleason Scores and incidence of metastasis.

## Methods

### Cell culture, antibodies and tumor samples

A benign prostatic hyperplasia cell line (BPH-1 cells) (generated by Hayward et al. [25] and initially obtained as a gift from P. Narayan, University of Florida) are routinely maintained in our laboratory in DMEM containing 10% FBS and antibiotic/antimycotic. BPH-1 cells were co-transfected with the ORFs of human LIMK1 or LacZ cloned in pIND vector and PVRxR plasmid (Invitrogen) to establish stable cell lines (BPHL and BPHLacZ) as described earlier [3]. Stable cells resistant to G418 (Invitrogen) (500 mg/ml) and Zeocin (Invitrogen) (50 ng/ml), and expressing LIMK1 or LacZ upon induction with ponasterone A (ecdysone analog) were selected and maintained in DMEM/10%FBS/antibiotic/antimycotic. In some cases, cells were induced with ponasterone A (Invitrogen) (5 *μ*M) for 24 h prior to harvest. Anti-LIMK1 (Transduction Laboratories), anti-α-tubulin (Sigma) and anti-glyceraldehyde-3-phosphate dehydrogenase (Biogenesis) monoclonal antibodies, and anti-γ-tubulin (Sigma) polyclonal antibodies were used for immunoblotting, immunofluorescence and flow cytometry. Anti-GAPDH antibodies were used in immunoblots to monitor GAPDH expression as an internal control. A low-density tissue microarray (TMA) of prostate tumors containing 50 prostate tumors and 3 uninvolved prostate tissues was obtained from IMGENEX (Histo-Array, IMGENEX 2.0 mm core diameter) [26]. The array provided by the manufacturer includes pathological reports and histories of metastasis for individual tumor tissues. The array was stained with anti-LIMK1 antibodies using immunohistochemistry. The anti-LIMK1 antibody is highly specific, does not cross react with LIMK2, and yields a 72 kD polypeptide band in western blots.

### Immunoblot, immunofluorescence and immunohistochemistry

Total cell lysates were resolved in SDS-PAGE and subjected to immunoblotting using anti-LIMK1 and anti-GAPDH antibodies. For indirect immunofluorescence, BPHLacZ and BPHL cells were plated on poly-L-lysine coated glass coverslips and induced with ponasterone A for 24 h prior to staining and in some cases treated with Taxol (10 nM) for various time points. Cells were permeabilized and either singly or dually stained with combinations of Alexa Fluor 488-conjugated phalloidin (Molecular Probes), anti-α-tubulin and anti-γ-tubulin antobodies. Alexa Fluor 488 or Cy3-conjugated anti-mouse or anti-rabbit antibodies (Molecular Probes) were used singly or in combination as the secondary antibody. Nuclei were stained using 4–6-diamidino 2-phenylindole, dilactic (DAPI, Molecular Probes). Fluorescent images were visualized in an epifluorescent microscope (Nikon TE300). The paraffin-embedded TMA was subjected to immunohistochemistry (IHC) using a pepsin-based antigen retrieval protocol (BioGenex, San Ramon, CA) and anti-LIMK1 antibodies. A biotinylated multi-link goat anti-immunoglobulin for mouse, rabbit, guinea pig and rat was used as the secondary antibody. Nuclei were counterstained with Hematoxylin QS (Vector Laboratories). Positive signals were detected by HRP-conjugated streptavidin and DAB as the chromagen (BioGenex Multi-Link kit).

### Immunostaining grade and statistical analysis

Immunostaining grades of the individual tumors in the array were assessed on the basis of a scale of 0 (no staining) to +4 (intense staining) [27][28]. The percentage of cells at each scale was estimated first and then the decimal-equivalent of the percentage was multiplied by the appropriate intensity score to obtain a weighted average of the intensity score [29]. This average, which will vary between 0 and 4, was referred to as the immunostaining score. To determine the discriminative ability of the cytoplasmic staining and nuclear staining scores, an approach based on receiver operating characteristic (ROC) curve analysis was used. ROC curves for the two measurements were calculated using the method of Pepe [30]. The cut points were chosen based on the region where the slope of the ROC curve was the greatest. Based on that cut point, a chi-squared test was used to test for association between clinical outcome and if the score exceeded the threshold. The clinical outcome considered was the presence or absence of metastases. This approach was taken relative to a t-test because of the relatively limited sample size and the fact that a difference in discriminative ability would manifest in a way other as a difference in mean expression.

### Cell synchronization and cell cycle analysis

BPHLacZ and BPHL cells were synchronized at the G1/S boundary using double thymidine (2 mM) treatment. Cells were treated with thymidine for 18 h, released from thymidine block for 8 h in fresh culture medium and blocked again with thymidine for 16 h. Cells were then released from the block in fresh medium containing bromodeoxyuridine (BrDU) (10 *μ*M). Ponasterone A was added 24 h before the last release of the thymidine block and cells were harvested at different times as specified. Cells were treated with nocodazole (0.1 μg/ml) for 18 h for synchronization at the G2/M boundary. Ponasterone A was added also at the same time. Cells were released from nocodazole block in fresh medium containing BrDU and collected at different time points as specified. Cells were reinduced every 24 h until they were harvested. Next, cells were stained with FITC-conjugated anti-BrDU antibodies and 7AAD using a BrDU labeling kit (Beckton Dickinson) according to manufacturer's specification and analyzed in a Flow cytometer (FACSCalibur, BD Biosciences). Raw data were analyzed using CellQuest and Modfit (BD Biosciences) software after elimination of aggregates. Cells present inside the gated area were used for calculation of percentage of cells in each phase.

### Taxol treatment

Asynchronous BPHLacZ and BPHL cells were induced with Ponasterone A and treated with Taxol (10 nM) for 24 h and 48 h. Cell cycle progression and DNA synthesis were analyzed using BrDU incorporation and propidium iodide staining. BrDU was added to monitor DNA synthesis 16 h before harvesting. Cells were processed for flow cytometry as described before. Growth of Taxol-treated cells was monitored using MTT [3-(4,5-dimethylthiazol-2-yl)-2,5-diphenyltetrazolium bromide] assays which produce formazan crystals upon cleavage of tetrazolium rings by mitochondrial dehydrogenase from viable cells using the protocol published elsewhere [31]. Values for MTT assays are presented as mean +SD. The significance of changes was determined by ANOVA (paired t-test) using Statview software (Abacus Concepts, Calabasas, CA). Statistical significance was established at *P *< 0.05.

## Results

### Altered cell morphology and abnormal centrosome number were noted in cells overexpressing LIMK1

**Figure 1:**
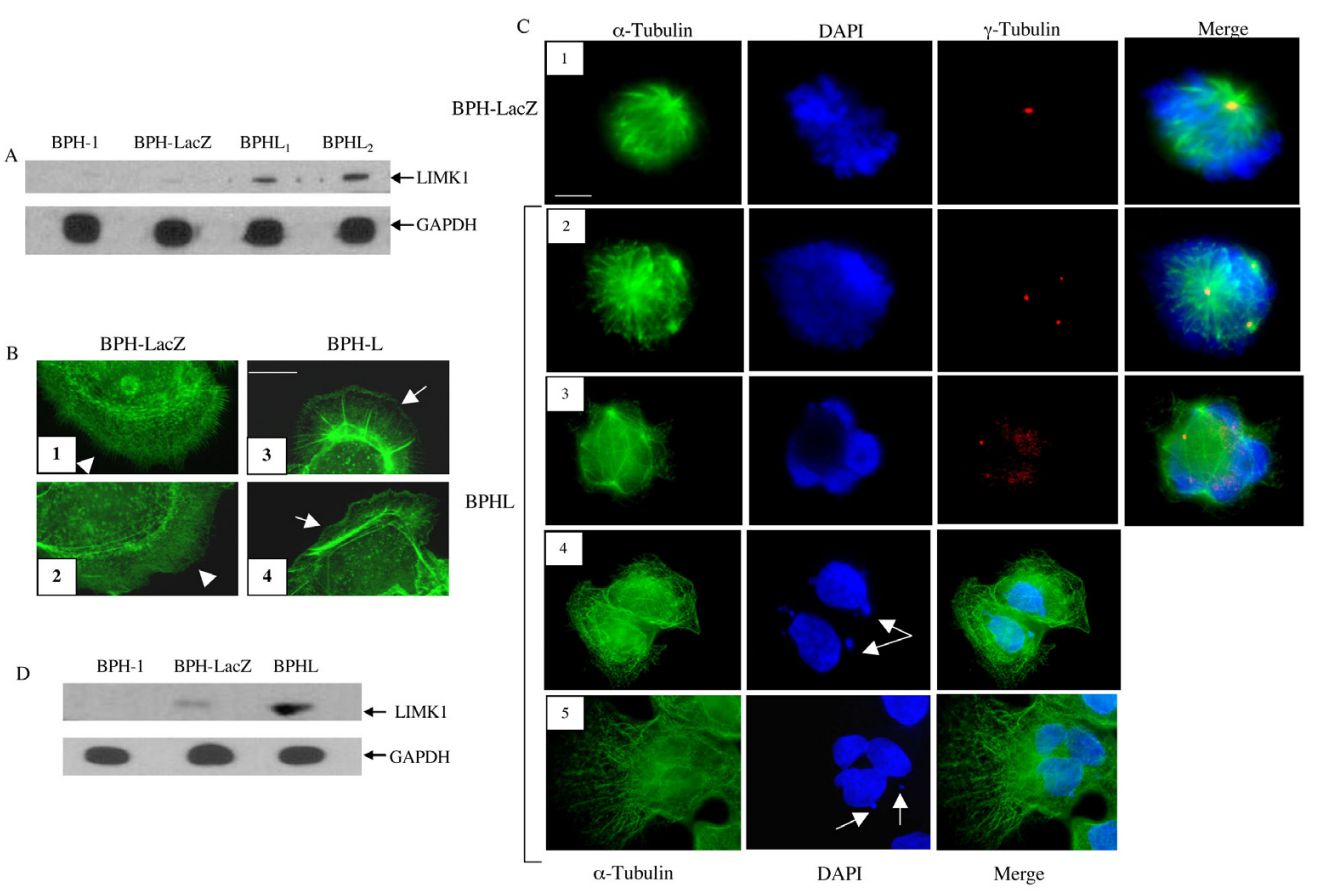
Expression of LIMK1 in BPH-1 cells and analysis of cell morphology. A: Western blot analysis of LIMK1 expression in BPH-1 parental cells, BPHLacZ and clones of BPHL cells showing increased expression of LIMK1 in BPHL cells. Expression of GAPDH was used as the loading control. B. Phalloidin staining of F actin showing appearance of lamellipodia and ruffling membranes with accumulation of actin in BPHL cells (arrows) (3, 4). BPHLacZ cells showed more microspikes but no lamelipodia with actin accunulation (arrowheads) (1, 2) (scale bar: 5 μM). C. Panel 1 demonstrates normal mitotic spindle and centrosome number with proper chromosomal alignment at the metaphase plate in BPHLacZ cells (scale bar: 5 μM). Because of the vertical position of the cell only one centrosome is visible. Panels 2 and 3 show abnormal spindle architecture with multiple centrosomes and altered chromosomal alignment in BPHL cells. Panel 4 demonstrates appearance of DAPI stained micronuclei in BPHL cells suggesting the presence of chromosomal instability (arrows). Panel 5 demonstrates the presence of multiple nuclei and micronuclei (arrows) in a cell. D: Western blot analysis of expression of LIMK1 in BPHL and BPHLacZ cells used for immunofluorescence analysis. Expression of LIMK1 in BPH-1 parental cells was used for comparison.

To understand the functional role of LIMK1 on cellular morphology, we investigated the effects of ectopic expression of LIMK1 in benign prostatic hyperplasia (BPH-1) cells. BPH-1 cells were chosen for these studies as these cells express low levels of LIMK1. Two stable clones of BPHL cells (BPHL1, BPHL2) showing significantly higher levels of expression of LIMK1 compared to the parental BPH-1 and BPHLacZ cells (Figure 1A) were selected and used for subsequent studies. Analysis of morphology of interphase cells following phalloidin staining for actin indicated appearance of lamellipodia at the cell periphery with accumulation of actin in BPHL cells (Figure 1B3 and 1B4), which was not seen in BPHLacZ cells (Figure 1B1 and 1B2). Immunofluorescence analysis also showed that the expressed LIMK1 was predominantly cytoplasmic in the interphase cells (data not shown).

To study the morphology of mitotic cells, we monitored the distribution of α and γ tubulins in transfected cells as components of microtubules and centrosomes respectively, by immunofluorescence analysis. Our results showed abnormal centrosome duplication and spindle assembly, such as multipolar spindles and abnormal chromosomal alignment at the equatorial plate in mitotic BPHL cells. Appearance of abnormal/disorganized spindles and multiple centrosomes were noted in 12% of the cells undergoing mitosis (Figure 1C2 and 1C3). None of these abnormalities was noted in mitotic BPHLacZ cells (Figure 1C1). Furthermore, 9–10% of the BPHL cells exhibited smaller DAPI stained chromosomal masses presumably micronuclei (Figure 1C4 and 1C5). Presence of multinucleated cells (Figure 1C5) also was noted in BPHL cells (5%), which express increased amounts of LIMK1 (Figure 1D).

### LIMK1 expression transiently arrested cells at G1/S phase

**Figure 2:**
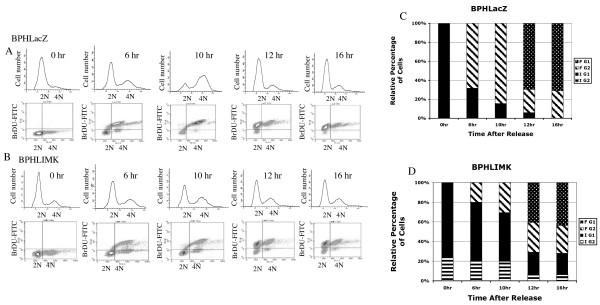
Flow cytometric analysis of progression of cells synchronized at the G1/S boundary. A, B: Upper panel demonstrates two-parameter histogram of distribution of BPHLacZ cells (A) and BPHL cells (B) in G1 (2N) and G2 (4N) phases at different times after release from thymidine block. Lower panel shows dot plots of BrDU incorporation in these cells as they progress through the S phase. Appearance of BrDU stained cluster of cells in G1 (upper left quadrant) at 12 h indicates entry of cells to the next cycle. C, D: Graphical representation of the percentage distribution of BPHLacZ cells (C) and BPHL cells (D) in initial G1 (IG1) (lower left quadrant), initial G2 (IG2) (lower right quadrant), final G1 (FG1) (upper left quadrant) and final G2 (FG2) (upper right quadrant) at different time points after release. Data shows temporary arrests of BPHL cells in G1/S phase. Similar profile was obtained in two separate experiments.

The effect of increased expression of LIMK1 on cell cycle progression specifically, progression of cells through the G1/S phase were evaluated next. We used induced BPHL and BPHLacZ cells synchronized at the G1/S boundary using a double thymidine block. Replication patterns and DNA contents of the cells released from the thymidine block were monitored by continuous incorporation of BrDU and staining with 7AAD at different time points after release (Figure 2). Analysis of DNA contents indicated that the percentage of the cells synchronized at the G1/S boundary (0 h) (Initial G1, IG1) was similar (81.3% BPHLacZ, 72.7%BPHL) for both cell types but a substantial number of BPHL cells (22.7%) were sluggish in progression and remained in the G2 phase of the previous cycle (initial G2 or IG2) as determined by the higher DNA content (Figure 2C and 2D). At 6 h after release, almost 70% of the BPHLacZ cells had duplicated their DNA as detected by BrDU incorporation, and moved to G2 phase (Final G2 or FG2) (Figure 2A and 2C), whereas only 17% of the BPHL cells duplicated their DNA and progressed to FG2 (Figure 2B and 2D). A gradual decrease in the percentage of BPHLacZ cells in IG1 during next 16 h and concurrent increase in the percentage of cells in FG2 reaching a peak at 10 h demonstrates progression of G1/S cells to the G2/M phase. After 10 h, the percentage of BPHLacZ cells in FG2 began to decrease and the anti- BrDU-FITC stained cluster of cells started to accumulate in G1 of the next cycle (Final G1, FG1) (Figure 2A and 2C). The complete transition of BPHLacZ cells with a maximum number of cells from IG1 to FG2 and then to FG1 was achieved at 16 h after release of the thymidine block (Figure 2A and 2C). Interestingly, no distinct FG2 peak for BPHL cells could be noted at any time. Also, about 20% of the BPHL cells remained stationary in IG1 as indicated by the lack of anti-BrDU incorporation in these cells. As a result, while 69% of the BPHLacZ cells progressed to FG1 only 40% of the BPHL cells entered G1 of the next cycle (FG1) (Figure 2D). The overall sluggish progression of BPHL cells indicated a transient arrest of cells at G1/S phase following induction of expression of LIMK1; this was not observed in BPHLacZ cells.

### Expression of LIMK1 delayed progression of cells through G2/M phase

**Figure 3:**
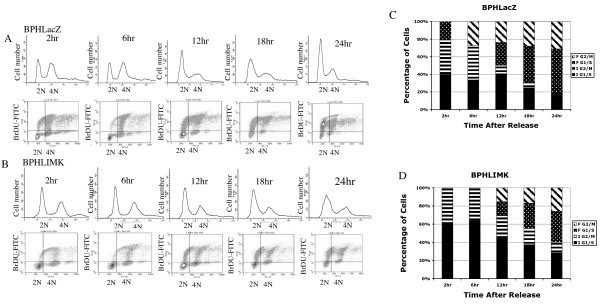
Profile of cell cycle progression of G2/M synchronized cells. A: Upper panel: Two-parameter histogram of the distribution of BPHLacZ cells in G2/M (4N) and G1/S (2N) at different time points after release from nocodazole block. Lower panel: Dot plots of BrDU incorporation in these cells as they progressed through the S phase of the current cycle and the next cycle. B: Upper panel: Allocation of BPHL cells in G1 and G2 phases following release from G2/M block showing an overall sluggish transition of G2/M cells to G1. Lower panel: Incorporation of BrDU in G2/M synchronized BPHL cells shows transition of cells through the S phase. Note low uptake of BrDU by cells with 2N DNA content. C, D: Percentage distribution of BPHLacZ cells (C) and BPHL cells (D) in IG1, IG2, FG1 and FG2. Note slow progression of the subset of BPHL cells in G2/M (checkered bar). Data presented here is a representative of two separate experiments.

Cells synchronized at the G2/M boundary were used next to determine the effect of LIMK1 on progression of cells through the G2/M phase. We monitored progression of Ponasterone A induced cells for 24 h after release from 18 h nocodazole block. Two hrs after release from the nocodazole treatment, 40% of the total BPHLacZ cells remained in IG1, 40% of the cells were synchronized and arrested in IG2 and 20% of the cells started progressing through the S phase and accumulated in FG1 as evident from the BrDU incorporation (Figure 3A and 3C). Between 6 h to 18 h after release, IG1 cells progressed through the S and then G2/M phases, whereas cells arrested at G2/M phase cycled to the G1 phase of the next cycle (FG1). Almost all G2/M arrested BPHLacZ cells (IG2) entered the next cycle after release and 80% of the total cells were distributed in FG1, S and FG2 phases at 24 h (Figure 3A and 3C). At 2 h after release, 60% of the total BPHL cells remained in IG1 and 40% of the cells were in IG2 phases. No distinct BrDU incorporated cell population could be detected as FG1 cells, which started progressing through the S phase (Figure 3B and 3D). At 12 h, 43% of the cells were in IG1 and did not incorporate BrDU, 21% of the cells were in IG2, whereas 14% cells were in each of the FG1 and FG2 phases. Cells designated as FG2 cells incorporated BrDU and progressed through the S phase. At 24 h, approximately, 30% of the BPHL cells were in the IG1 phase, and had not incorporated BrDU and 10% of the cells were in the IG2 phase (Figure 3B and 3D). About 30% of the cycling cells were in FG1 and 25% cells were in FG2 at 24 h.

### LIMK1 prevented paclitaxel-mediated G2/M arrest and multinuclearity

**Figure 4:**
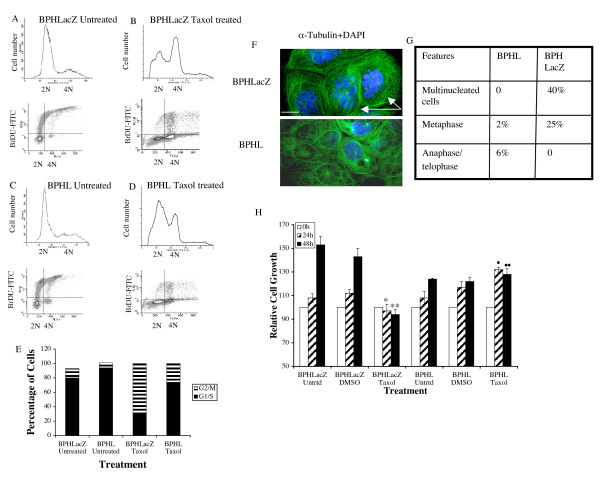
Effect of LIMK1 expression on Taxol treatment. A, C: Upper panel: Two-parameter histogram of cell cycle profile of asynchronous BPHLacZ (A) and BPHL (C) cells. Lower panel: BrDU incorporation by asynchronous BPHLacZ and BPHL cells. B, D. Upper panel: Distribution of BPHLacZ cells (B) and BPHL cells (D) in different phases of the cell cycle following Taxol-treatment for 24 h showing accumulation of BPHLacZ cells but not BPHL cells in G2/M containing 4N DNA. Lower panels show limited incorporation of BrDU in both cell types. E. Percentage distribution of BPHLacZ and BPHL cells in G1/S and G2/M phases with or without treatment with Taxol. F. Microscopic analysis of morphology of BPHLacZ and BPHL cells following Taxol treatment (scale bar: 5 μM). Arrows indicate multinucleated giant BPHLacZ cells as noted by DAPI staining. G: quantitative analysis of multinucleated cells, cells in metaphase and cells in anaphase/telophase following Taxol treatment of BPHLacZ and BPHL cells. Five hundred cells were counted for percentage analysis. Data represents the profile obtained from two separate experiments. H. MTT assays of the metabolic activity of cells treated with Taxol or DMSO. Equal amounts of cells were seeded and harvested at 24 and 48 h. Data shows a significant reduction in metabolic activity representing inhibition of cell proliferation in BPHLacZ cells following Taxol treatments. BPHL cells showed proportionately increased metabolic activity with or without Taxol treatment. Data represents Mean+ SD of three separate experiments (* *P *< 0.001 compared to BPHLacZ DMSO 24 h; **P < 0.02 compared to BPHLacZ DMSO 48 h; • p < 0.01 compared to BPHLacZ Taxol 24 h; •• *P *< 0.05 compared to BPHLacZ Taxol 48 h). Untreated BPHL cells showed a slower rate of growth compared to BPHLacZ cells.

Because our previous experiment indicated delayed transition of BPHL cells from G2/M to G1, we intended to study the involvement of LIMK1 in mitotic process following paclitaxel (Taxol) treatment. Ponasterone A induced transfected cells were treated with Taxol for 24 h, and mitotic arrests were assessed by monitoring 7AAD staining of the DNA contents. We have labeled cells continuously with BrDU (16 h) in order to examine progression of cells beyond G1. While asynchronous BPHLacZ and BPHL cells showed similar profiles of G1, S and G2/M cells and BrDU incorporation (Figure 4A and 4C histograms and dot plots) Taxol treatment induced a G2/M arrest in BPHLacZ cells as evident by a larger peak of 4N cells (Figure 4B histogram and dot plot, lower right quadrant) and low incorporation of BrDU (Figure 4B dot plot upper left quadrant). A much smaller peak of 4N cells were noted in Taxol treated BPHL cells indicating a lesser number of cells in G2/M phase (Figure 4D histogram and dot plot lower right quadrant). Taxol treated BPHL cells were accumulated in G1 phase as indicated by a larger peak of 2N cells ((Figure 4D, histogram and dot plot, lower left quadrant). Quantitative analysis of distribution of cells showed that a much higher percentage of BPHLacZ cells (70%) were in G2/M phase compared to BPHL cells (26%) following Taxol treatment (Figure 4E).

Immunofluorescence analysis of α tubulin and DAPI staining showed that a significant number of Taxol-treated BPHLacZ cells were with multiple nuclei (40%) (Figure 4F and 4G) and about 25% of the cells were arrested in metaphase (Figure 4F). No multinucleated BPHL cell was detected following Taxol treatment. Only a small percentage of these cells were in metaphase and anaphase (2% and 6%, respectively) (Figure 4F and 4G). MTT-based growth analysis indicated that Taxol treatment induced growth retardation in BPHLacZ cells within 24 h compared to untreated (vehicle) cells. No inhibition of growth of Taxol treated BPHL cells was observed at 24 h compared to DMSO treated cells. Growth patterns of untreated BPHLacZ and BPHL cells showed an overall slower proliferation rate of BPHL cells compared to BPHLacZ cells (Figure 4H).

### Expression of LIMK1 was associated with prostate cancer

**Figure 5:**
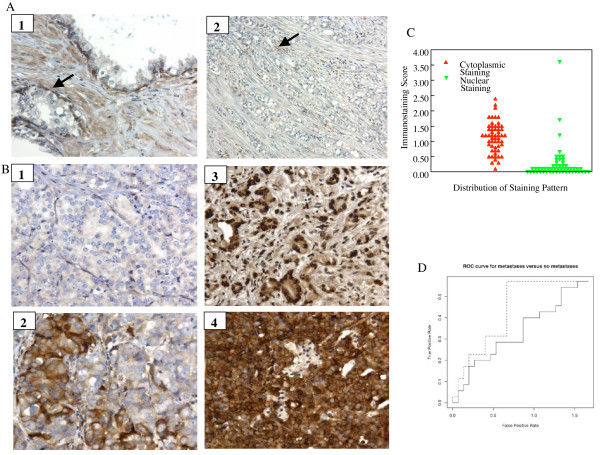
Expression of LIMK1 in prostate tumors. A: Panel 1 demonstrates the strong staining of basal cells of benign prostatic glands with LIMK1 (solid arrow) (X400). In panel 2, there is a very weak staining of a poorly differentiated prostatic adenocarcinoma (X400). Moderate staining of scattered apparent smooth muscle cells can be identified in the stroma immediately adjacent to malignant cells (solid arrow). B: Panel 1 shows very weak staining of the cytoplasm of prostate cancer with no nuclear staining (X400). Panel 2 demonstrates no staining to strong nuclear staining of prostatic adenocarcinoma. The cytoplasmic staining varies from weak to strong (X400). Panel 3 demonstrates very strong nuclear staining for LIMK1 for prostatic adnocarcinoma and moderate to strong cytoplasmic staining (X400). Panel 4 demonstrates very strong cytoplasmic staining for LIMK1 for prostatic adenocarcinoma. There is also variable nuclear staining of prostatic adenocarcinoma for LIMK1, which varies from no staining to moderate staining. C. Comparison of immunostaining scores between cytoplasmic and nuclear staining. D. Receiver operating characteristic (ROC) analysis of cyoplasmic (solid line) and nuclear staining (dotted line) in relation to incidence of metastasis or no metastasis. ROC curve for nuclear staining showing a shift towards the y-axis indicates a better association of nuclear staining with the incidence of metastasis compared to cytoplasmic staining.

To assess whether there is any correlations between expression of LIMK1 and prostate cancer we have evaluated the staining profile of LIMK1 in a number of prostate tumors from patients with history of metastasis using immunohistochemistry. The median age of patients at the time of diagnosis was 68 years (range 44–88 years). According to the TNM classification of tumors, 62% of the patients showed history of either lymph node or distant metastasis at the time of surgery or biopsy. Also, 88% of the tumors exhibited Gleason Scores of 7 or above. Tumor samples without any clinical history of distant or lymph node metastasis had Gleason Scores between 6 to 10 (Table 1). A blinded immunohistochemical analysis indicated a differential pattern of LIMK1 staining in tumors and surrounding stromal areas. A variable cytoplasmic staining of LIMK1 from very weak (Figure 5B1) to strong (Figure 5B4) was noted in poorly differentiated prostate adenocarcinomas. Nuclear staining for LIMK1 also varied from no staining to strong staining in these samples. The pattern of staining of the stroma was quite variable. In general, there was no staining of the inflammatory cells. The staining of smooth muscle cells was both cytoplasmic and nuclear with usually one staining pattern clearly predominating. Expression of LIMK1 was higher in the stromal cells in close proximity of the tumors in majority of the cases compared to the stroma surrounding BPH. In uninvolved normal glands of the prostate, the nuclei and cytoplasm of basal cells stained moderately to strongly for LIMK1 while there is weak staining for LIMK1 of the cytoplasm of the luminal cells. The strength and pattern of staining of basal cells is similar to or stronger than staining of some of the smooth muscle cells of the stroma, which stain with LIMK1 (Figure 5A1 and 5A2). Evaluation of immunostaining grades in tumor areas indicates a relatively broader range of cytoplasmic staining compared to nuclear staining (Figure 5C). Statistical analysis of the immunostaining grades indicated that cytoplasmic staining has no association with incidence of metastasis (Chi-squared p-value = 0.4), although the nuclear staining has almost significant association (Chi-squared p-value = 0.07) (Figure 5D).

**Table 1:** Distribution of tumors based on the Gleason Scores and history of metastasis with respect to nuclear and cytoplasmic staining of LIMK1

Gleason Scores	# Samples	Incidence of metastasis	Cytoplasmic and Nuclear Staining	Cytoplasmic Staining Only	No Incidence of metastasis	Cytoplasmic and Nuclear Staining	Cytoplasmic Staining Only
6	6	0	0	0	6	1	5
7	9	8	7	1	1	0	1
8	11	7	3	4	4	2	2
9	6	5	3	2	1	1	0
10	18	13	8	5	5	1	4

**Figure 6:**
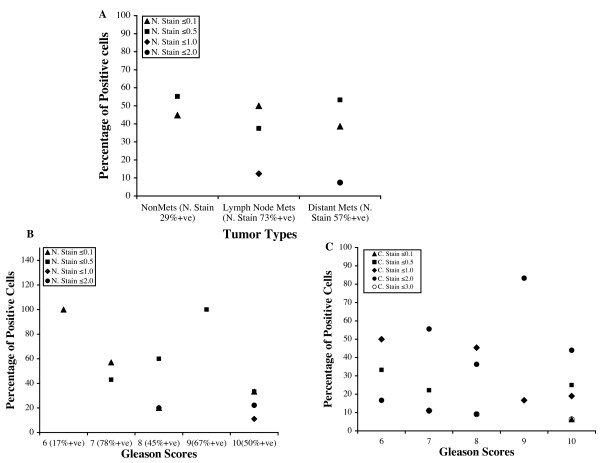
Analysis of immunostaining intensity in tumor cells with respect to incidence of metastasis and Gleason Scores. A: Relative intensity of the nuclear stain with respect to percentage of metastatic and nonmetastatic tumors. Only the cells showing positive nuclear staining of LIMK1 were used for the analysis. Distant Mets: Prostate tumors with history of distant metastasis; Lymph node Mets:Prostate tumors with history of lymph node metastasis. NonMets: Prostate tumors without any history of metastasis. % positive: Percentage of tumors showing nuclear stain. B: Relative immunostaining scores of nuclear stain in percentage of LIMK1 positive tumors with respect to Gleason Scores. C. Relative immunostaining scores of cytoplasmic stain in percentage of tumors with respect to Gleason Scores.

Analysis of relative intensity of nuclear stain between groups of metastatic (57% positive) and nonmetastatic (27% positive) tumors indicated higher immunostaining scores in a larger percentage of metastatic tumors compared to nonmetastatic ones (Figure 6A). When immunostaining grades were correlated with Gleason Scores, positive nuclear staining was observed in 17% of the tumors with Gleason Scores of 6 whereas, 45% to 78% of the tumors with 7 or higher Gleason Scores exhibited positive nuclear staining (Figure 6B, Table 1). Increased nuclear staining intensity was also noted in tumors with higher Gleason Scores (Figure 6B). Although all tumor samples exhibited positive cytoplasmic staining for LIMK1 in the luminal cells a propensity of increased intensity of LIMK1 staining and higher Gleason Scores could be noted (Figure 6C).

## Discussion

In this study, the consequence of overexpression of LIMK1 in prostate epithelial cells, and any association of LIMK1 with prostate cancer have been assessed. Here we provide evidence that an elevated expression of LIMK1 induces abnormal centrosomal multiplication and generation of multipolar spindles in BPH-1 cells. Disorganization of spindles in BPHL cells may occur as a result of altered stability of microtubules. In a recent study, LIMK1 has been shown to be involved in microtubule destabilization [32]. Appearance of multinucleated cells and micronuclei, markers for genetic instability, also were evident in response to increased expression of LIMK1. Although centrosomal defects that often occur at the advanced stages of cancer are believed to contribute to genetic instability by altering fidelity of chromosomal segregation during mitosis, centrosomal defects also occur concurrently with chromosomal abnormalities and cytological changes in early stages of cancer [33-36]. Pihan et al demonstrated that progressive dysfunction of centrosomes and misallocation of centrosomes occur during prostate cancer progression and such changes increase with increasing Gleason Score in invasive cancer [37]. It is possible that the centrosome and spindle defects induced by increased expression of LIMK1 may play a role in phenotypic alterations in BPHL cells. Such changes may also cause BPHL cells to become highly invasive compared to naturally noninvasive BPH-1 parental cells. Our earlier studies showed that expression of LIMK1 made BPHL cells highly invasive compared to BPHLacZ cells [3].

Our results on cell cycle analysis indicated an altered cell cycle progression in BPHL cells, which showed a distinct but transient arrest of cells at the G1/S phase and delayed progression of cells through the G2/M phase. These findings are in concurrence with the idea that chromosomal abnormalities are intimately involved in generation of cell cycle defects, which frequently become a phenotypic characteristic of advanced cancers [38]. Although LIMK1 expression led to a transient arrest in G1/S phase it did not inhibit cell proliferation as evident from BrDU incorporation, which is in contrast to the earlier reports on the inhibitory effect of LIMK1 on NIH3T3 cell proliferation [39]. The transient nature of G1 arrest implies a temporary block that possibly depends on expression of one or more key proteins. It is possible that the block is released when concentrations of these proteins reach a threshold level.

Our experiments showing a delayed G2/M transition following increased expression of LIMK1 suggest the possibility of cytokinesis defects rendered by increased concentration of LIMK1. This observation supports the earlier report showing that expression of LIMK1 in HeLa cells induced cytokinesis defects [13,14]. Nonetheless, this defect did not arrest cells permanently in the mitotic phase. G2/M synchronized cells progressed to G1/S and incorporated BrDU at a later time showing resumption of the cell cycle progression. This observation suggests that the concentration of LIMK1 needs to be tightly regulated for proper progression of mitosis and cytokinesis. These observations are supported by our MTT assay data, which showed an overall slower growth rate of BPHL cells compared to BPHLacZ cells. Expression of LIMK1 also promoted resistance to Taxol induced metaphase arrest, cell growth retardation and appearance of multinucleated cells as a possible predisposition for apoptosis [40]. It is speculated that the microtubule destabilizing effect of LIMK1 interferes with the Taxol-induced microtubule stabilization and inhibits normal cellular response to Taxol. However, the exact mechanism whereby LIMK1 expression confers resistance to Taxol induced cellular responses is not clear and is a subject for further study.

Our studies on expression of LIMK1 in prostate tumors showed that all tumor samples were positive for weak to strong cytoplasmic expression of LIMK1 compared to no or very weak expression in the luminal cells of uninvolved prostate glands. Expression of LIMK1 was noted in basal cells of uninvolved tissues by IHC. Because basal cells are highly proliferative in nature, they may require expression LIMK1 for rapid cell growth. Furthermore, increased expression of LIMK1 in stromal cells in close proximity to the tumor areas in majority of the tumor samples may have a functional significance in phenotypic changes associated with advanced tumors. It has been documented that reciprocal interactions between stroma and tumor epithelium play important roles in acquisition of metastatic phenotypes by the prostate tumors [41,42]. Our studies also indicated a possible correlation between the extent of nuclear or cytoplasmic expression of LIMK1 in the luminal cells and Gleason Scores. Analysis of immunostaining scores also showed an almost significant association between increased expression of nuclear LIMK1 and history of metastasis. Nonetheless, our study strongly suggests an association between expression of LIMK1 and prostate cancer.

To summarize, this study provides evidence that an elevated expression of LIMK1 generates chromosomal instability and cell cycle defects. This report also shows that overexpression of LIMK1 is associated with prostate cancer. The level of expression of LIMK1 achieved in our experiment was either parallel to or less than that noted in advanced prostate tumors and in PC3 prostate cancer cells; this suggests that over expression of LIMK1 in prostate tumors may suffice to elicit the biological effects noted here. Furthermore, overexpression of LIMK1 conferred resistance to Taxol-mediated mitotic arrest and multinucleated giant cell formation. Taxol, an agent widely used for treatment of cancer, works through the inhibition of cell cycle by activation of mitotic checkpoints. Expression of LIMK1 may deregulate mitotic checkpoints in cancer cells whereby it can promote development of resistance of advanced prostate cancer to taxenes. Further studies are required to assess the relevance of LIMK1- mediated deregulation of cell cycle in progression of prostate cancer.

## Conclusion

LIMK1 is involved in regulation of actin cytoskeleton and microtubule dynamics and as a result, plays important roles in cell division and cell behavior. Our studies imply that the concentration of LIMK1 needs to be tightly regulated for proper cell cycle progression and that up regulation of LIMK1 may contribute to tumor progression through altered cell cycle pattern and chromosomal defects.

## Competing interests

The author(s) declare that they have no competing interests.

## Authors' contribution

MD and RC performed experiments and analyzed data. DJ and WEG performed the Gleason scoring and immunostaining grading in tumor samples and analyzed data. DG performed statistical analysis of the immunostaining grades. RC and WEG wrote the manuscript and all authors read and approved the final version of the manuscript.
